# Construction of a high-density mutant library in soybean and development of a mutant retrieval method using amplicon sequencing

**DOI:** 10.1186/s12864-015-2079-y

**Published:** 2015-11-26

**Authors:** Mai Tsuda, Akito Kaga, Toyoaki Anai, Takehiko Shimizu, Takashi Sayama, Kyoko Takagi, Kayo Machita, Satoshi Watanabe, Minoru Nishimura, Naohiro Yamada, Satomi Mori, Harumi Sasaki, Hiroyuki Kanamori, Yuichi Katayose, Masao Ishimoto

**Affiliations:** Agronomics Research Center, National Institute of Agrobiological Sciences, 2-1-2 Kannondai, Tsukuba, Ibaraki 305-8602 Japan; Present address: Gene Research Center, Faculty of Life and Environmental Sciences, University of Tsukuba, 1-1-1, Ten-nodai, Tsukuba, Ibaraki 305-8574 Japan; Faculty of Agriculture, Saga University, 1 Honjo-machi, Saga, 840-8502 Japan; Present Address: Soil Science and Plant Nutrition Division, National Agriculture and Food Research Organization Agricultural Research Center, 3-1-1, Kannondai, Tsukuba, Ibaraki 305-8666 Japan; Present Address: Faculty of Agriculture, Niigata University, 8050, Ikarashi 2-no-cho, Nishi-ku, Niigata, 950-2181 Japan; Nagano Vegetable and Ornamental Crops Experiment Station, 1066-1, Soga, Shiojiri, Nagano, 399-6461 Japan

**Keywords:** EMS mutagenesis, Mutant library, High resolution melting, Next generation sequencing, Mutation density, Amplicon sequencing, Nextera technology, HiSeq, MiSeq, DNA pooling

## Abstract

**Background:**

Functions of most genes predicted in the soybean genome have not been clarified. A mutant library with a high mutation density would be helpful for functional studies and for identification of novel alleles useful for breeding. Development of cost-effective and high-throughput protocols using next generation sequencing (NGS) technologies is expected to simplify the retrieval of mutants with mutations in genes of interest.

**Results:**

To increase the mutation density, seeds of the Japanese elite soybean cultivar Enrei were treated with the chemical mutagen ethyl methanesulfonate (EMS); M2 seeds produced by M1 plants were treated with EMS once again. The resultant library, which consisted of DNA and seeds from 1536 plants, revealed large morphological and physiological variations. Based on whole-genome re-sequencing analysis of 12 mutant lines, the average number of base changes was 12,796 per line. On average, 691 and 35 per line were missense and nonsense mutations, respectively. Two screening strategies for high resolution melting (HRM) analysis and indexed amplicon sequencing were designed to retrieve the mutants; the mutations were confirmed by Sanger sequencing as the final step. In comparison with HRM screening of several genes, indexed amplicon sequencing allows one to scan a longer sequence range and skip screening steps and to know the sequence information of mutation because it uses systematic DNA pooling and the index of NGS reads, which simplifies the discovery of mutants with amino acid substitutions.

**Conclusions:**

A soybean mutant library with a high mutation density was developed. A high mutation density (1 mutation/74 kb) was achieved by repeating the EMS treatment. The mutation density of our library is sufficiently high to obtain a plant in which a gene is nonsense mutated. Thus, our mutant library and the indexed amplicon sequencing will be useful for functional studies of soybean genes and have a potential to yield useful mutant alleles for soybean breeding.

**Electronic supplementary material:**

The online version of this article (doi:10.1186/s12864-015-2079-y) contains supplementary material, which is available to authorized users.

## Background

Soybean [*Glycine max* (L.) Merr.] is a major source of the world’s protein, oil, and animal feed [1]. The reference genome sequence (975 Mb) [2] comprises 54,175 predicted protein-coding loci. The functions of most of these predicted genes have not been clarified. A mutant library with a high mutation density would be helpful for obtaining such functional evidence. Soybean has a paleopolyploid genome and nearly 75 % of predicted soybean genes are present in multiple copies due to two duplication events at 13 and 59 mya [3]. Compared with diploids, polyploid species can better withstand higher mutation densities because of compensation by other genome copies [4, 5], as additional gene copies may mask the phenotypic effect of a mutation. However, RNA-seq analysis of transcriptional divergence of duplicated genes by neo-functionalization has shown that approximately 50 % of duplicated genes were differentially expressed in soybean [6]. Retention of the ancestral function by one of the duplicated genes and acquisition of a novel function by the other [7, 8] may result in phenotypic changes despite the high genetic redundancy in the soybean genome.

Gamma ray is the most commonly used physical mutagens in plant mutation breeding [9] and induces deletions [10]. One of the reverse genetics approaches, induction of point mutations by chemical mutagenesis, known as TILLING (targeting-induced local lesions in genomes) [11], has been widely used to discover the biological functions of sequenced genes and to develop novel alleles associated with specific traits in several crop species such as maize [12], wheat [4], rice [13], sorghum [14], soybean [15], tomato [16], and canola [17]. Ethyl methanesulfonate (EMS), N-nitroso-N-methylurea (NMU), and ethyl nitrosourea (ENU) constitute 64 % of all agents used for chemical mutagenesis (reviewed in [18]). In soybean, EMS is commonly used, and diverse mutant phenotypes have been reported (reviewed in [19]). Mutation densities of up to 1/140 kb detected by TILLING have been reported in EMS- or NMU-induced mutant population [15]. According to Anai [20], the mutation rate, 1/769 kb, which was detected from mutant population by repeated EMS treatment of soybean increased from the mutation rate, 1/2500 kb, which was detected from another mutant population by a single treatment of EMS.

A heteroduplex mismatch cleavage assay based on mismatch-specific endonuclease Cel I is the most established method to detect point mutations [11, 21], but in this method the reaction conditions depend on the target region [22]. High resolution melting (HRM) analysis [23, 24] has been recently applied as an alternative simple, rapid, and inexpensive method for mutant discovery in tomato [25] and wheat [26], although its accuracy depends on the quality of PCR, resolution of the instruments, and the choice of fluorescent dyes [24]. Some types of base changes (such as C to G or A to T) are more difficult to detect by HRM than other types due to the small differences in melting temperatures [27]. Sanger sequencing [28] is generally required as the final step of mutation screening to confirm the base changes and whether the mutation causes an amino acid substitution.

Next generation sequencing (NGS) technologies provide accurate and rapid detection of mutations. NGS platforms such as Roche 454 pyrosequencing [29], sequencing-by-synthesis [30], SOLiD sequencing [31], and Ion Torrent sequencing [32] were released from 2005 to 2010. The HiSeq 2000 (Illumina, San Diego, CA, USA) became the standard of high-throughput sequencing; it uses sequencing-by-synthesis chemistry. With the widespread use of HiSeq 2000, Illumina launched an alternative platform MiSeq, compact bench-top sequencers. Sequencing with MiSeq, which is also based on sequencing-by-synthesis chemistry, has lower throughput (up to 15 Gb yield per run of 300 bp reads) than HiSeq 2000 (up to 600 Gb yield per run of 100 bp), but it is cheaper, faster, and easier to use [33]. However, accurate detection of single nucleotide variants (SNV) requires sequencing coverage of 20× to 30× for a diploid genome [34], and re-sequencing of large populations is still expensive. An alternative, cheaper method is sequencing of PCR amplicons in targeted regions, so-called amplicon sequencing [17, 35, 36]. A combination of a pooling strategy and sequencing in the targeted region by NGS platforms has been examined [34, 37]. The drawbacks and limitations of sequencing pooled samples by NGS include difficulties in distinguishing low-frequency alleles from sequencing errors, the need to adjust the DNA concentration, occurrence of misalignment to the reference sequence due to high error rates, and short sequence reads [37]. Nextera (Illumina), a library construction method referred to as tagmentation, combines DNA fragmentation and DNA tagging in a single reaction of transposase-mediated adaptor insertion [38]. This method enables convenient and quick genomic library construction for high-throughput sequencing [38, 39]. Development of such cost-effective and high-throughput commercial products based on NGS technologies may simplify the retrieval of mutants with mutations in the target genes.

In the present study, we constructed a high-density mutant library of soybean, which provide a potential to yield new alleles for soybean breeding. An efficient method of mutant discovery in the library, which uses DNA pooling and a new commercial dual indexing system for the amplicon sequencing by NGS, is described.

## Results and discussion

### Mutant library

**Fig. 1:**
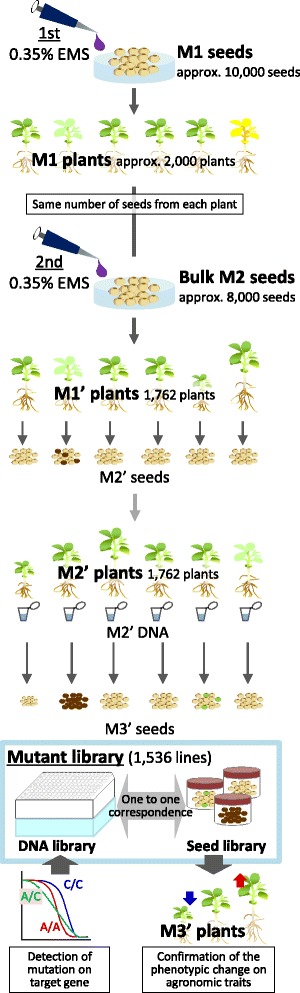
Outline of the construction of the EMS-induced mutant library. Seeds were treated with a chemical mutagen (0.35 % EMS). To increase the mutation density, bulk M2 seeds from approximately 2000 M1 plants were treated again with 0.35 % EMS and 8000 M2 seeds used to grow M1’ plants; the generation after the second mutagen treatment was called M1’ to discriminate it from the initial M1 generation. M2' seeds were collected each from 1762 M1' plants. A total of 1762 M2' plants were grown and used to extract DNA. Twenty-six potential out-crossing plants were removed, resulting in a total of 1736 M2’ plants. The resultant library consisted of DNA derived from 1536 M2’ plants; 1437 of which produced seeds. Seeds from each line were stored with their corresponding DNAs

Using seeds of a Japanese elite soybean cultivar Enrei, we developed a mutant library with a high mutation density to identify useful novel alleles for breeding (Fig. 1). M1 seeds were treated with 0.35 % EMS; M2 seeds were collected from four per M1 plant and bulked from approximately 2000 M1 plants, treated in a similar way to the first treatment in order to increase mutation density, and used to grow M1’ plants. The generation after second EMS treatment was called M1’ to discriminate it from the initial M1 generation. Populations were kept small and of the similar size in every generation. Since contamination of DNA from out-crossing plants critically impacts on estimation of mutation density and identification of mutant, out-crossing plants was carefully examined by simple sequence repeat (SSR) analysis. After all, 26 potential out-crossing plants were removed in order to preserve genetic integrity. The resultant library consisted of DNA derived from 1536 M2’ plants; 1437 of which produced M3' seeds. Seeds from each line and their corresponding DNA samples were stored as a mutant library.

### Phenotypic variations

**Fig. 2:**
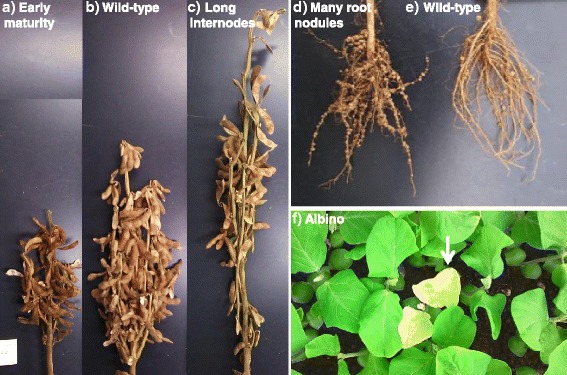
Mutant phenotypes observed in the M2’ plants. Wild-type: (**b**), (**e**); Mutant phenotypes: (**a**) Early maturity, (**c**) Long internodes, (**d**) Many root nodules, (**f**) Albino

Phenotypic variations were observed in the following traits: albino, the density of leaf pubescence, flowering time, flower color, internode length, the number of nodules, maturity time, pod size, seed size, and seed color. Some particular phenotypes distinct from wild-type cultivar Enrei are shown in Fig. 2 and listed in Table 1. The frequency of typical mutations, such as albino phenotype, was considerably higher in our library than in previously reported soybean mutant populations [40, 41]. The frequency of the albino phenotype mutations (~4.9 %) in a population mutagenized by using a combination of 45 kR gamma rays and 0.2 % EMS [42] was similar to 4.4 % in the present library, suggesting that mutations can be accumulated by repeated mutagenesis. The frequency of the dwarf phenotype in the present library, 1.6 %, was higher than 0.26 % reported by Karthika and Lakshmi [40] for M2 plants mutagenized with 0.2 % EMS. The frequency of root nodule mutants, 0.6 %, was also greater than 0.04 % reported in the same cultivar by Akao and Kouchi [41] despite their use of a higher EMS concentration (0.5 % compared with 0.35 % in our study). The frequency of dwarf and semi-dwarf mutants in the present library, 9.1 %, was considerably higher than 1.34 % in a wheat population mutagenized with 0.8 % EMS [4] and 2.89 % in common bean (0.6 % EMS) [43]. Similarly, the frequency of albino mutants, 4.4 %, was higher than 0.27 % in wheat [4], 1.7 % in tomato [44], and 1.53 % in common bean [43]. The frequencies of other crop mutants with higher concentration (0.6–1.0 % EMS) were even less than half the frequency compared to 0.35 % EMS in our library. Thus, various mutant phenotypes to be potential target for forward genetics approach were frequently observed in the present library in comparison with other high-mutation-density mutant libraries.

**Table 1:** Frequency of typical mutant phenotypes detected in the library

Phenotype description	Number of plants	Frequency (%)**
Albino (medium - heavy)*	76	4.4
Rugose leaves, dwarf*	11	0.6
Rugose leaves, semi-dwarf*	20	1.2
Rugose leaves, normal	31	1.8
Dwarf	17	1.0
Semi-dwarf	110	6.3
Early flowering	46	2.6
Early maturity	29	1.7
White flowers	6	0.3
Violet flowers	8	0.5
Short internodes	2	0.1
Long internodes	3	0.2
Narrow leaves	8	0.5
Low-density pubescence	3	0.2
Big primary leaves	6	0.3
Early defoliation	1	0.1
Easy lodging	1	0.1
Many leaves	1	0.1
Long peduncles	1	0.1
Many rootlets	2	0.1
Many root nodules	10	0.6
No root nodules	1	0.1
Many pods and early pod maturity	1	0.1
Wide pods and early maturity	2	0.1
Large seeds	27	1.6
Deep yellow seed coat	4	0.2
Light brown seed coat	2	0.1

*Most plants produced no or few seeds

**Frequency (%) was calculated from number of mutant for each phenotype divided by 1736 M2’ plants

**Fig. 3:**
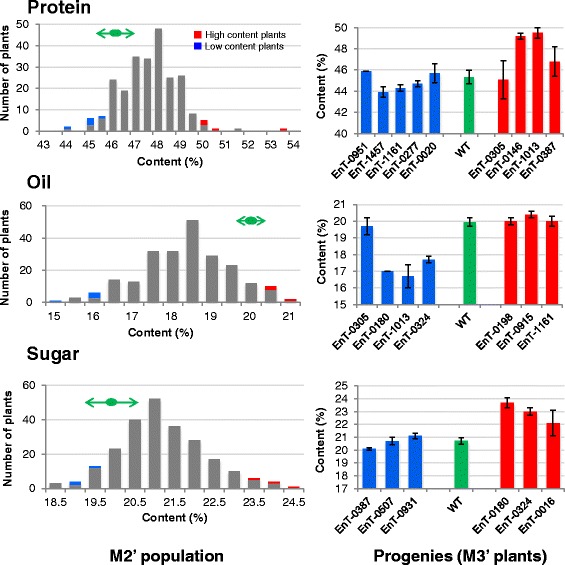
Variations in protein, oil, and sugar content among seeds harvested from M2’ plants and its progenies. Left histograms and right bar graphs indicate variations in protein, oil, and sugar content of the seeds harvested from M2’ plants and the progenies (M3’ plants), respectively. High content plants (red) and low content plants (blue) in M2’ population were re-evaluated at M3’ plants. In the left histograms, mean and variation for wild-type plants (WT) are indicated by green ellipses and double-headed arrows, respectively

Protein, oil, and sugar content in seeds harvested from M2’ plants is shown in Fig. 3. In comparison with wild-type plants (protein: 46.3 ± 0.2 %, oil: 19.7 ± 0.0 %, sugar: 19.8 ± 0.2 %), distribution and average seed protein (47.3 ± 1.3 %) and sugar content (20.9 ± 1.1 %) in the library was significantly shifted toward higher values, whereas that of oil content (18.1 ± 1.2 %) was shifted toward lower values (*p* < 0.01). The ranges of protein content (43.7–53.2 %) and oil content (14.6–20.8 %) became considerably wider than those of the protein content (40.8–46.4 %; [45, 46]) and oil content (17.3–19.4 %; [46]) reported in some Japanese soybean cultivars and less than those of the protein and oil content (26.2–56.0 %; 11.5–31.4 %, respectively) reported to 4400 germplasm collection [47]. A negative correlation was observed between protein and oil content (*r* = −0.60, *p* < 0.01), protein and sugar content (*r* = −0.42, *p* < 0.01), and oil and sugar content (*r* = −0.39, *p* < 0.01). A strong negative correlation between seed protein and oil content is commonly recognized [48–50]. Generally, phenotype reproducibility of several M2’ plants with the high protein content, high sugar content and the low oil content was confirmed in their M3’ progenies (Fig. 3). In contrast, phenotype reproducibility as for M2’ plants with low protein, low sugar and high oil content was unclear in M3’ progenies. In soybean seeds, 7S and 11S globulins comprise approximately 70 % of total storage proteins [51–53]; two QTLs that determine the ratio of 7S globulin to 11S globulin have been identified [54]. Although many genomic regions are related to the content of seed protein [48], oil, and sugar [55], actual genes that control the content of these components have not been identified yet. Mutant lines with the maximal changes in seed protein, oil, and sugar content observed in the present study may be suitable materials for a smart forward genetics approach such as MutMap [56] that allows rapid causal gene identification for the mutant by whole-genome resequencing of pooled DNAs from segregating progenies with phenotypic differences.

### Mutation density

To estimate the number of base changes per line, 12 arbitrarily selected mutant lines were re-sequenced. The cleaned short read sequences were mapped to the Williams 82 reference genome assembly. The aligned read depth ranged between 14.3 and 25 fold; genome coverage ranged between 93.6 and 94.3 % (Table 2). Common base changes shared by different mutants were treated as polymorphic sites between Enrei and Williams 82 and were filtered out. The resultant number of base changes in each mutant line ranged from 8970 to 21,861 with an average of 12,796 mutations per line (Table 2). More than 90 % of base changes induced by EMS and NMU are known to be G to A and C to T transitions [18]. As expected, those transitions were most frequently observed in the mutant lines (Table 2), but the rates were lower and ranged from 53 to 77 %. The frequency of T to A and A to T transversions, the second most frequently observed group of mutations, ranged from 12 to 33 %. Among 153,554 base changes in all lines (Table 2), 106,480 G > A and C > T transitions was predominant (69.3 %) followed by 27,306 T > A and A > T transversions (17.8 %), 7626 T > C and A > G transition (5.0 %), 5981 G > T and C > A transversions (3.9 %), 4578 A > C and T > G transversions (3.0 %) and 1583 C > G and G > C transversions (1.0 %). The transition to transversion ratio was 2.89 (114,106/39,448). Interestingly, the rates for G > A and C > T transitions, T > A and A > T transversions and the transition to transversion ratio were quite similar with that reported in Tomato (69.3 %, 12.9 % and 2.9, respectively; [57]).

**Table 2:** Mutations in 12 lines detected by using whole-genome re-sequencing analysis

Line name	Depth of coverage	Genome coverage (%)	Number of base changes	Type of base change												Amino acid substitutions	Misssense mutations	Nonsense mutations	Distance between base changes (kb)**
				G > A	C > T	T > A	A > T	T > C	A > G	G > T	C > A	A > C	T > G	C > G	G > C				
EnT-0263	16.8	94.2	9934	3776	3481	635	718	266	300	202	206	135	117	60	38	573	543	30	95.7
EnT-0394	14.3	94.0	15579	5494	6493	1023	985	298	266	262	245	170	194	85	64	950	899	51	61.0
EnT-0442	17.0	94.1	8970	3612	3179	541	541	231	229	146	159	129	107	56	40	564	535	29	106.0
EnT-0541	23.1	93.9	15627	5666	5638	1120	966	459	452	338	334	237	220	103	94	823	785	38	60.8
EnT-0790	16.9	93.9	9366	3391	3366	661	609	262	252	171	212	155	160	65	62	514	485	29	101.5
EnT-0964	19.3	94.3	21861	8099	7688	1980	1972	397	373	374	324	265	263	61	65	1303	1246	57	43.5
EnT-1045	21.5	93.9	9172	2428	2926	1217	996	349	272	205	235	195	212	84	53	442	426	16	103.6
EnT-1079	18.0	94.0	14074	5376	5167	1057	919	292	297	271	247	183	150	69	46	902	865	37	67.5
EnT-1197	15.2	94.0	13782	4516	4877	1383	1234	353	295	269	289	194	227	79	66	796	756	40	69.0
EnT-1610	25.0	93.8	9481	2536	2874	1220	1048	388	338	250	226	220	227	91	63	439	425	14	100.3
EnT-1619	19.4	93.8	13274	5187	4122	1077	1269	273	291	318	250	191	170	66	60	729	686	43	71.6
EnT-1634	20.5	93.6	12434	3306	3282	1997	2138	331	362	228	220	233	224	63	50	684	646	38	76.5
Average	18.9	94.0	12796	4449	4424	1159	1116	325	311	253	246	192	189	74	58	727	691	35	74.3
Percentage of each type of base change*				34.8 %	34.6 %	9.1 %	8.7 %	2.5 %	2.4 %	2.0 %	1.9 %	1.5 %	1.5 %	0.6 %	0.5 %				

*The percentage of each type of base change was calculated from a total each type of base change in 12 mutants divided by all base changes

**The distance between base changes was calculated from a total number of base changes per plant and the size of chromosome-scale assembly of the soybean genome (950,068,807 bp)

**Fig. 4:**
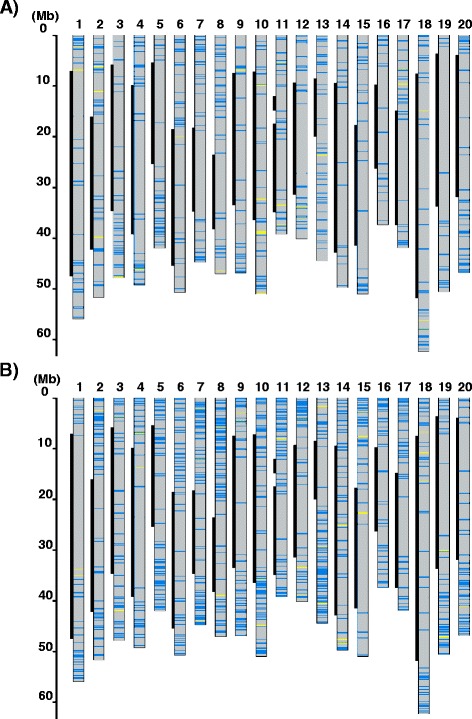
Distribution of mutations affecting the amino acid sequence on 20 chromosomes of two mutant soybean lines. Blue and yellow lines on the chromosomes of two mutant lines, EnT-1634 (**a**) and EnT-0964 (**b**), indicate missense and nonsense mutations, respectively. The black line on the left of each chromosome indicates the pericentromeric region with lower gene densities than surrounding euchromatic region

On average, 727 base changes per line had the potential to cause amino acid substitutions based on the Glyma_189 annotation (Table 2). Among the base changes, 691 and 35 were missense and nonsense mutations, respectively. The distribution of mutations affecting the amino acid sequence on 20 chromosomes of two mutant lines, EnT-1634 and EnT-0964, is shown in Fig. 4. The mutation density was estimated to be approximately 1 mutation per 74 kb (range, 43–106 kb), which was much higher than expected. So far, the highest density, up to 1/140 kb induced by either 0.5 % EMS or 2.5 mM NMU, was reported in soybean [15]. The mutation density of a mutant library in this study was approximately 2 times higher than that of population. Indeed duplicated mutations should be considered to be included in the present library due to EMS treatment twice. Nonsense mutation is known to cause gain-of-function allele as well as loss-of-function allele depend on the mutation position in human disease study (reviewed in [58]). Therefore, the present library rich in nonsense mutation has a potential to contain novel mutant allele and provide alternative useful phenotype exceed known trait variations. Although too many mutations per plant may mask the intended mutant phenotype and hamper phenotype characterization, the present library with a small population size is practical for handling, screening, and maintenance; it will provide a tool for functional studies and yield novel mutant alleles for soybean breeding.

### Mutant retrieval methods

**Fig. 5:**
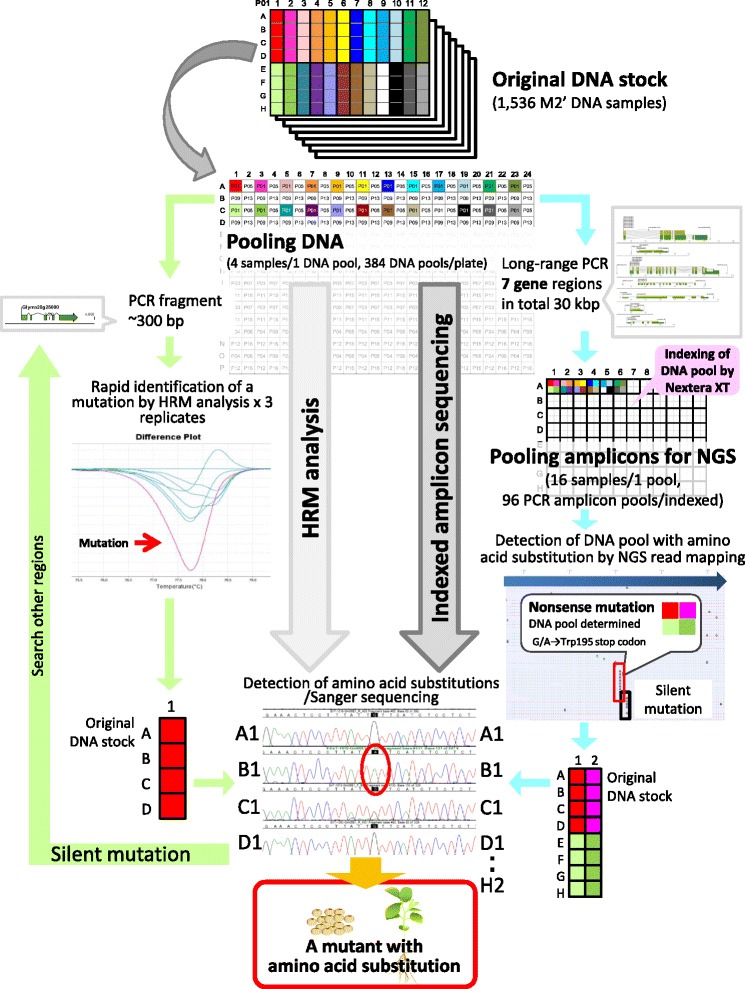
Mutant discovery by using HRM and indexed amplicon sequencing. DNA extracted from M2’ plants was preserved as the original DNA stock in 96-well plates. The DNA pool in a 384-well plate (four samples per pool) was used for both methods. After a mutation was detected by HRM analysis, base changes in four original DNA samples were confirmed by direct sequencing. If the mutation was found to be silent, HRM analysis and direct sequencing of other regions were performed. In indexed amplicon sequencing, 7 target gene regions (1.3–7.5 kb, 30.3 kb in total) were amplified by long-range PCR. The amplicons of four samples were further pooled. The 96 samples were indexed by using a transposome-based Nextera XT Index kit. Bulk read data for all 96 DNA pools were obtained from Miseq and mapped onto the reference sequences of target genes after classification of the DNA pool by using indices. Base changes at high frequency in many reads were treated as a mutation and were filtered by using a Glyma_189 gene annotation to exclude mutations that did not lead to amino acid substitutions. Based on the information from DNA pool classification with indices, the base change and the plant in which it occurred could be determined by direct sequencing of each of the 16 original M2’ DNA samples. Amplicon sequencing using NGS allows rapid and effective detection of DNA pools containing mutations that cause desirable functional amino acid substitutions

Two strategies tested here for the identification of mutants with amino acid substitutions in the mutant library are shown in Fig. 5. Advances in NGS methodologies provide cost-effective commercial products for rapid identification of sequence variations across the genome. However, the traditional method based on Sanger sequencing is still widely used to confirm the accuracy of sequences obtained by NGS. Therefore, both strategies include mutation confirmation by direct sequencing as the final step (Fig. 5). Primers for HRM analysis need to amplify short fragments of up to ~300 bp within a target gene. According to instruction manual for HRM analysis (Applied Biosystems, Foster City, CA, USA), at least three repetitions are recommended to determine DNA pool containing a mutation in PCR product. In the present study, the four original DNA samples from the DNA pool with a different HRM signature are subjected to a second round of HRM analysis or direct sequencing. If the identified mutation causes no amino acid substitution, it is necessary to return to the primer design step to amplify an alternative region.

Using our second strategy, indexed amplicon sequencing, we analyzed seven target genes (1.3–7.5 kb; 30.3 kb in total) amplified by long-range PCR in each DNA pool (Fig. 5). Amplicons of four samples were further pooled; this arrangement is critical to simplify re-sampling for confirmation of the mutation by direct sequencing at the final step. Dual indexing of the 96 pooled samples and sequencing library preparation were conducted with a transposome-based Nextera XT Index Kit (Illumina). Advantages of the kit are the simple procedure for fragmentation and tagmentation. The NGS read data classified by using the indexes were mapped separately onto the reference sequences of target genes. Base changes observed in more than 2 % of the aligned reads were treated as a mutation; the mutations were filtered by using Glyma_189 gene annotation to exclude mutations not leading to amino acid substitutions. Since each DNA pool with a candidate mutation can be identified by sequences of the dual indexes, it was possible to detect candidate mutations without a screening step such as HRM analysis. For plants with mutations that cause amino acid substitutions, the sequences were simultaneously confirmed by direct sequencing.

### HRM

The results of HRM analysis of four coding DNA sequence of Glyma20g25000 (locus *Ln*) (Additional file 1: Figure S1), which encodes a transcription factor responsible for leaflet shape [59], are shown as a model case in Table 3. Twenty-six plants with base changes were identified in the mutant library. Since the EMS treatment was performed twice, duplicated mutations (in a homozygous or heterozygous state, Table 3) derived from M1 plants are present in the library. After removal of such duplicated mutations, the mutation density in the library was estimated as at least 15.7 mutations per 1 kb from the number of unique 18 mutations in the 1144-bp region. The 18 independent mutations were observed within the 1144-bp region in total, most of which were either G to A or C to T transitions except for a A to T mutation in a line EnT-1312. Among all 18 mutations, ten were missense mutations, but no nonsense mutations were detected.

**Table 3:** Mutations in Glyma20g25000 detected by high resolution melting analysis

Line name	Target region	Amplicon size (bp)*	Base change	Mode of mutation	Chromosome	Position (bp)	Amino acid substitution
EnT-0541	Ln ex1	332	G > A	hetero	Gm20	34688627	Met1Ile
EnT-0685	Ln ex1	332	C > T	hetero	Gm20	34688652	Leu10Phe
EnT-1168	Ln ex1	332	G > A	homo^**^	Gm20	34688672	Syn
EnT-0112	Ln ex1	332	G > A	homo^**^	Gm20	34688682^a^	Asp20Asn
EnT-0044	Ln ex1	332	G > A	hetero	Gm20	34688682^a^	Asp20Asn
EnT-1589	Ln ex1	332	G > A	hetero	Gm20	34688682^a^	Asp20Asn
EnT-1376	Ln ex1	332	G > A	hetero	Gm20	34688686^b^	Gly21Asp
EnT-0621	Ln ex1	332	G > A	hetero	Gm20	34688686^b^	Gly21Asp
EnT-1048	Ln ex1	332	C > T	homo^**^	Gm20	34688696^c^	Syn
EnT-1306	Ln ex1	332	C > T	homo^**^	Gm20	34688696^c^	Syn
EnT-0987	Ln ex1	332	C > T	homo^**^	Gm20	34688713	Ser30Phe
EnT-0160	Ln ex1	332	C > T	hetero	Gm20	34688719	Ser32Phe
EnT-0634	Ln ex2	231	G > A	homo^**^	Gm20	34689275^d^	Gly40Ser
EnT-0749	Ln ex2	231	G > A	homo^**^	Gm20	34689275^d^	Gly40Ser
EnT-0439	Ln ex2	231	C > T	hetero	Gm20	34689313	Syn
EnT-1084	Ln ex2	231	G > A	hetero	Gm20	34689360	Gly68Glu
EnT-1281	Ln ex3	114	C > T	hetero	Gm20	34689704	Ala102Val
EnT-1312	Ln ex3	114	A > T	hetero	Gm20	34689710	His104Leu
EnT-0601	Ln ex4	467	G > A	homo^**^	Gm20	34690049	Syn
EnT-0155	Ln ex4	467	G > A	hetero	Gm20	34690058	Syn
EnT-0687	Ln ex4	467	C > T	homo^**^	Gm20	34690175^e^	Syn
EnT-0862	Ln ex4	467	C > T	homo**	Gm20	34690175^e^	Syn
EnT-1619	Ln ex4	467	G > A	hetero	Gm20	34690246	Gly213Asp
EnT-1265	Ln ex4	467	C > T	homo^**^	Gm20	34690247^f^	Syn
EnT-0510	Ln ex4	467	C > T	hetero^***^	Gm20	34690247^f^	Syn
EnT-0383	Ln ex4	467	C > T	hetero	Gm20	34690247^f^	Syn

*The amplicon size does not include primer sequences

**Base changes in a homozygous state probably occurred in M1 plants

***Base changes in a heterozygous state are probably derived from the same M1 plants as those labeled with **

Syn indicates synonymous site at which a base substitution does not cause an amino acid substitution

Superscripts a to f indicate that the mutation was duplicated in plants labeled with the same letter

Although HRM analysis detected 26 candidate lines for Glyma20g25000 in total (Table 3), Sanger sequencing showed that the mutations in 11 lines led to synonymous amino acid substitutions, which have no effect on protein structure. While excess amount of sequence confirmation by Sanger sequencing will be required to confine the candidates to meaningful mutations, simple and cost-effective HRM analysis still provides an easy-to-use platform for mutation screening.

### Indexed amplicon sequencing

**Fig. 6:**
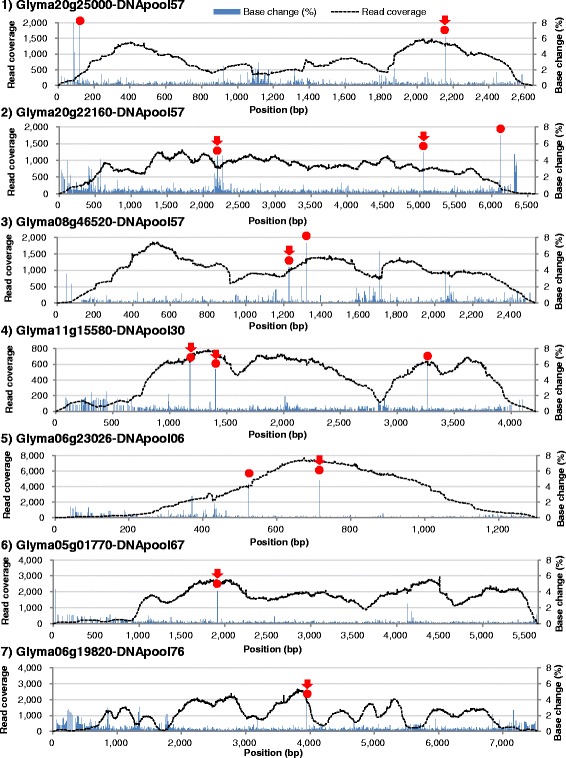
Distribution of read coverage across seven amplicons and observed base changes in the DNA pool. Base change positions called as mutations are shown by red circles. Red arrows indicate confirmed by Sanger sequencing, respectively

Seven genes of a total length of 30.3 kb were analyzed by indexed amplicon sequencing (Table 4, Additional file 1: Figure S2). The total length of amplicons was adjusted based on the sequence output of MiSeq v2 Chemistry so that the read coverage for each sample would fall in a range between 50× and 100×. In addition to Glyma20g25000 described above, Glyma08g46520, encoding a cytochrome P450 that participates in soyasaponin biosynthesis [60]; Glyma06g23026 (maturity locus *E1*), encoding a transcription factor [61]; Glyma11g15580, a pseudo-response regulator family gene involved in photoperiod response [62]; and Glyma20g22160 (maturity locus *E4*), encoding a photoreceptor [7], Glyma05g01770 and Glyma06g19820, which encode betaine-aldehyde dehydrogenases related to soybean fragrance [63], were included in the analysis. The total read length and paired-end read counts were 6,010,609,329 bp and 12,021,219 before trimming and 4,054,408,786 bp and 11,190,596 after trimming, respectively. After de-multiplexing the bulk of the NGS read data into 96 pools by using the indexes, read mapping on the reference gene sequences was conducted separately pool-by-pool. The total mapped read length and count for the 96 pools were 4,015,703,044 bp (99.1 %) and 22,130,334 (Table 4), respectively. The read counts for each gene varied from 1,044,416 to 6,977,332 depending on the length of the gene. However, relatively large variation was observed for read coverage depending on the DNA pool and sample (Table 4) as well as sample well location (Additional file 1: Figure S3). For instance, the total read number for the shortest gene Glyma06g23026 was higher than expected, and total read counts per DNA pool (average: 6097 reads) and per sample (average: 381 reads) were the highest among the examined genes. In contrast, read coverage for Glyma11g15580 was the lowest for the number of reads per DNA pool (average: 518 reads) and per sample (average: 32 reads). Since one DNA pool contained 16 samples, at least a read with a base change among 32 reads was required to detect one sample with a point mutation in a heterozygous state. Sufficient read coverage is required to distinguish between sequencing errors and low-frequency mutant alleles because of the relatively high error rates of NGS reads [37]. The average read coverage per pool and per sample obtained in this study (Table 4) might be sufficient for this purpose. However, the read coverage per pool was distributed non-uniformly across amplicons (Fig. 6). Read coverage varied by position and generally was lower in AT-rich regions and at both ends of the amplicons. The percentage of base changes increased in such low-coverage regions because of an increased contribution of sequencing errors in the limited number of reads. Although the threshold for calling a mutation was set to 2 % of base changes, not all base changes above 2 % were called as mutations. Many base changes were excluded because the read coverage was lower than the required minimum read coverage (at least 96 reads required), or because of their absence in either forward or reverse read. The base changes that met these criteria were treated as mutations (Fig. 6, red circles).

**Table 4:** Read coverage and mutations in seven genes identified by using indexed amplicon sequencing

Gene locus*	Amplicon size (bp)	Consensus length (bp)	Total read counts for 96 DNA pools	Read coverage per sample			Base changes	Type of base change					Amino acid substitutions	Misssense mutations	Nonsense mutations	Total number of base changes expected per M2' plant**	Distance between base changes (kb)***
				Average	Minimum	Maximum		G > A	C > T	T > A	A > T	Others					
Glyma06g19820	7539	7519	3476540	54	13	137	88	46	30	1	4	7	30	29	1	7224	132
Glyma05g01770	5643	5625	3844251	80	5	172	67	26	35	1	2	3	26	25	1	7348	129
Glyma08g46520	2531	2514	1476507	69	18	166	77	31	36	2	6	2	44	43	1	18828	50
Glyma06g23026	1304	1294	4156094	381	33	1188	32	17	12	1	0	2	12	10	2	15188	63
Glyma20g22160	6390	6370	6977332	130	35	276	132	61	56	1	3	11	72	69	3	12785	74
Glyma11g15580	4214	4190	1155194	32	8	124	101	36	35	0	3	27	28	27	1	14833	64
Glyma20g25000	2648	2623	1044416	47	10	137	64	26	26	2	9	1	21	21	0	14958	64
Total	30269	30135	22130334				561						233	224	9	(av.) 14269	(av.) 67.0
Percentage of each type of base change****								43.3 %	41.0 %	1.4 %	4.8 %	9.4 %					

*Gene locus names are indicated according to gene models in the Glyma_189 assembly (v1.1)

**The total number of base changes per M2' plant was calculated from a total number of base changes in the library estimated from the amplicon size and the size of chromosome-scale assembly of the soybean genome (950,068,807 bp) and then divided by the total number of plants (1536)

***The distance between base changes was calculated from the size of the amplicon divided by the total number of base changes found in the library

****The percentage of each type of base change was calculated from a total each type of base change in seven genes divided by all base changes

A total of 561 unique base changes were identified in the seven genes by indexed amplicon sequencing (Table 4). Base changes at the same site were counted as one base change. In the results for several mutants, G to A and C to T transitions (84.3 %) and T to A and A to T transversions (6.2 %) were most frequently observed. Most mutations occurred in one sample (322 mutations, 57 %) but some were observed in two samples (64 mutations, 11 %), there samples (18 mutations, 3 %) and four or more samples (11 mutations, 2 %). The large variation in the number of base changes (from 32 to 132) was due to the variation in the length of target gene regions from 1304 to 7539 bp. The number of base changes per M2' plant were estimated by the base change frequency in each gene, and ranged from 7224 to 18,828 (average: 14,269). Similarly, the distance between base changes differed by the genes and ranged from 50 to 132 kb (average: 67.0 kb). The mutation density and the number of mutations per plant obtained by indexed amplicon sequencing were very similar to the results of whole-genome re-sequencing (range: 43–106 kb, average: 74 kb, Table 2). Among 561 base changes, 224 were missense mutations and nine were nonsense mutations (Table 4). Since 60 % of the base changes caused synonymous amino acid substitutions, which have no effect on protein structure, indexed amplicon sequencing can effectively confine the candidates to meaningful mutations. All nonsense mutations were confirmed by Sanger sequencing (data not shown); most of them may be useful for functional studies. Confirming the potential impact of missense mutations with the aid of the database of catalytic domain structures will be needed.

In the present study two strategies, HRM analysis and indexed amplicon sequencing, were used to screen the mutant library. Although the overall mutation density in the library and individual plants was determined by whole-genome re-sequencing analysis based on 12 mutants, all of mutation for the targeted genes used for indexed amplicon sequencing remained unknown in 1536 M2’ mutants. To validate whether sequence variations identified by these methods provide reliable information about mutations in the whole library, the results of screening of five genes (total length of 3.5 kb) were compared with the results of HRM analysis (Table 5). In addition to the results for Glyma20g25000 (Table 3), several regions of the four genes were analyzed by HRM. All 107 base changes obtained from two methods were confirmed by Sanger sequencing as the final step. HRM analysis detected 62.5 % of the 107 base changes, whereas indexed amplicon sequencing identified 90.3 %. Most sequence variations observed in the NGS reads by indexed amplicon sequencing reflect mutations well in the library in comparison with the HRM analysis data. The failure of indexed amplicon sequencing to detect the remaining 10 % of mutations was mainly due to low read depth and a uniform threshold for calling a mutation. These parameters may affect the discovery of mutations. Therefore, adjustment threshold for calling a mutation and increasing the read depth in samples is required for each gene and can improve consistency. The reasons for the lower discovery rate by HRM analysis seem to be that pooling four DNA samples is not suitable for any target regions and/or that A to T transversions are difficult to detect by HRM analysis due to their small differences in T_m_ [27].

**Table 5:** Comparison of mutation detection by high resolution melting analysis and indexed amplicon sequencing

Gene locus	Length compared (bp)	All base changes obtained from two methods	Base changes common to both methods	HRM analysis only	Index amplicon sequencing only	Percentage of mutations detected by HRM analysis (%)	Percentage of mutations detected by index amplicon sequencing (%)
Glyma20g25000	1144	37	14	4	19	48.6	89.2
Glyma08g46520	487	22	13	1	8	63.6	95.5
Glyma06g23026	305	12	7	0	5	58.3	100.0
Glyma20g22160	1187	24	12	4	8	66.7	83.3
Glyma11g15580	380	12	7	2	3	75.0	83.3
Total	3503	107	53	11	43	62.5	90.3

A longer sequence range of indexed amplicon sequencing in comparison with HRM can be scanned all at once. Multiple gene copies impede mutation discovery in polyploid plants because it is difficult to design specific primers that can identify polymorphisms among copies of the same gene [17]. In contrast to HRM, in the proposed method of indexed amplicon sequencing it is not difficult to find specific primer pairs that can amplify a specific PCR amplicon because the choice of the target region is very flexible. Furthermore, the proposed method allows one to skip screening steps because it uses DNA pooling and a commercial dual indexing system, which enables simple and effective detection of plants with functional mutation and can be used as an alternative method for mutant screening. Since enlargement of present library toward 6000 lines are currently underway, severe adjustment of balance between number of target genes, the sequence range, number of indices and pooling individuals will be required to screen more mutants in the near future. Further advances in detection sensitivity which allow the detection of single molecules by third-generation sequencing is already described [33], but this technology still developing. The development of more cost-effective and higher-throughput whole-genome sequencing technologies would provide accurate data about mutations in all mutants in our upcoming library.

## Conclusions

A soybean mutant library with a high mutation density was developed and various mutant phenotypes were observed at a high percentage in comparison with other previous soybean mutant libraries. A high mutation density (1 mutation per 74 kb) was achieved by repeating the EMS treatment. Since multiple mutations probably mask the mutant phenotype of interest, confirmation of a functional mutation by the progeny analysis of test-cross with a wild-type plant is required. The mutation density of our library is sufficiently high to obtain a plant in which a gene is nonsense mutated. Thus, our mutant library and the indexed amplicon sequencing will be useful for functional studies of soybean genes and have a potential to yield useful mutant alleles for soybean breeding.

## Methods

### Plant materials, mutagenesis, and growth conditions

Mutagenesis was performed according to Anai [20]. The overall scheme of mutagenesis, plant propagation, and mutant library generation is shown in Fig. 1. Approximately 10,000 seeds of Japanese soybean (*Glycine max* L. Merr. cv. Enrei) were soaked for 8 h in a 0.35 % EMS (Wako Pure Chemical Industries, Osaka, Japan) solution and then washed with running tapped water for overnight. Seeds were kept at 4 °C for 2 days prior to planting. M1 seedlings were grown in a vinyl house for 2 weeks and then transplanted to a field on the Hitachiomiya Campus of the National Institute of Agrobiological Sciences (NIAS) in Hitachiomiya, Ibaraki, Japan. The M1 plants (2000 individuals) were grown with an inter-row spacing of 70 cm and hill spacing of 10 cm. Four M2 seeds were collected from each M1 plant, resulting in a total of 8000 seeds, which were mutagenized again with 0.35 % EMS as above. These seeds were called M1’ (Fig. 1), and M1’ plants were grown as above. M2’ seeds were harvested from individual M1’ plants. The M2’ plants were grown as above except that the inter-row spacing was 80 cm and hill spacing was 30 cm and M3’ seeds were harvested at a field on the NIAS in Tsukuba, Ibaraki, Japan.

### Mutant evaluation

During the growing season, M2’ plants were examined for particular phenotypic changes that would distinguish them from wild-type ‘Enrei’. The number of days from sowing to first flowering (R1) and full maturity (R8) [64] was recorded if these dates were at least one week earlier than those of wild-type. Protein, oil, sugar, and moisture contents in more than 10 g of the intact seeds were measured by near-infrared spectroscopy with a Foss Infratec 1241 Grain Analyzer using the calibration model SO138111 Soybeans STM (FOSS North America, Eden Prairie, MN, USA). Seeds from 242 M2’ plants, M3’ progenies and wild-type plants were examined (maximum four M3’ progenies per M2’ plant). Transmission spectra were recorded at a wavelength range of 570–1100 nm. All values were recorded as means of three measurements per plant.

### DNA extraction

Total genomic DNA of M2’ plants was extracted from young fresh leaves (0.3 g) by using guanidine hydrochloride (Sigma-Aldrich, St. Louis, MO, USA) and proteinase K (QIAGEN, Valencia, CA, USA) according to Khosla et al. [65] with modifications. DNA concentration was measured with a Quant-iT™ PicoGreen dsDNA assay kit (Invitrogen, Carlsbad, CA, USA) according to the manufacturer’s manual on a VIIA™ 7 Real-Time PCR System (Life Technologies, Carlsbad, CA, USA) and adjusted to approximately 35 ng/μl. Two multiplex PCR reactions that amplified 14 soybean SSR markers (Additional file 2: Table S1) were used to exclude contamination due to outcrossing with other materials according to the method of Kongjaimun et al. [66] with minor modifications. DNA samples were then pooled (four samples per well of a 384-well plate) and the DNA pool was used as a template DNA for screening.

### Detection of mutations by whole-genome re-sequencing

Whole-genome re-sequencing was performed on a HiSeq™ 2000 platform according to the manufacturer’s instructions (Illumina) at Beijing Genomics Institute (Beijing, China). Briefly, a 100-bp paired-end library with an insert size of 500 bp was constructed by using genomic DNA from 12 M2’ plants following the TruSeq™ protocol (Illumina). Read mapping and variant detection were performed with CLC Genomics Workbench 6.5.2 (CLC Bio, Aarhus, Denmark). To reduce sequencing errors, stringent filtering was applied to the raw reads with the following parameters: adaptor trim, ambiguous limit two, quality limit 0.01, and removal of three 5'- and 3'-terminal nucleotides, discard read pairs with a minimum number of nucleotides less than 50 bp. The cleaned paired-end reads were mapped onto the soybean reference genome sequence (cultivar Williams 82, version Gmax_189; [3]) obtained from Phytozome FTP site [67] with the following parameters: no global alignment, auto-detect paired distances, mismatch cost two, deletion cost three, insertion cost three, length fraction 0.9, and similarity fraction 0.96. The complete genome sequences of soybean chloroplast (NC_007942) and mitochondrion (NC_020455) from the NCBI database [68] were also included as reference organelle genomes. SNV were identified in the aligned reads with probabilistic variant detection modules with the following parameters: ignore non-specific matches and broken read pairs, minimum read coverage five, variant probability 40 %, and presence in both forward and reverse reads required. Insertions and deletions were excluded from analysis. To remove variations of paralogous reads and variations between Williams 82 and Enrei, another SNV set was collected for each plant by using the same modules with the following relaxed parameters: non-specific matches and broken read pairs included, minimum read coverage two, variant probability 10 %, and maximum expected variants four. By comparing the sets from 12 M2’ plants to each other, a variant filter to distinguish unrelated variation from mutations was prepared based on a common frequency threshold of 20 %. Using this variant filter, a set of high-confidence single nucleotide changes that occurred because of EMS treatment was created for each plant. Position of mutations overlapping with known gene annotations and those resulting in amino acid changes were searched by using amino acid changes annotation module against the annotated version of Gmax_189 obtained from Phytozome FTP site [67].

### Primer design

Primers were designed on the basis of the reference soybean genome sequence by using Primer3 [69] with default parameters. To ensure specificity, primer sequences were searched against Gmax_189 to examine the number of potential binding sites, amplicon size and location by using Genome Tester [70] with default parameters until a single amplicon was obtained. For HRM analysis, 16 primers (Additional file 2: Table S2) were designed to amplify fragments shorter than 500 bp for Glyma20g25000, Glyma08g46520, Glyma06g23026, Glyma11g15580, and Glyma20g22160. For amplicon sequencing, seven primer pairs (Additional file 2: Table S3) were designed to amplify long fragments (1.3–7.5 kb) of Glyma05g01770 and Glyma06g19820 in addition to the above five genes.

### HRM analysis

Mutations were detected with a VIIA™ 7 Real-Time PCR System (Life Technologies). Reaction mixtures consisted of either 5 μl of MeltDoctor™ HRM master mix (Life Technologies), 2.5 μl of 10 μM forward and reverse primers, and 0.2 μl of the template DNA in a total volume of 10 μl, or 5 μl of 2× GoTaq® Colorless Master Mix (Promega, Madison, WI, USA), 2.5 μl of 10 μM forward and reverse primers, 0.003 μl of 5 mM SYTO® 9 green fluorescent nucleic acid stain (Life Technologies), and 0.2 μl of the template DNA in a total volume of 10 μl. For MeltDoctor™ HRM master mix, the PCR thermal cycler was programmed as follows: 1 cycle of initial denaturation at 95 °C for 10 min; 40 cycles of denaturation for 15 s at 95 °C, annealing for 30 s at 53 °C, and extension for 1.5 min at 60 °C. For GoTaq® Colorless Master Mix, the initial denaturation time was 2 min and denaturation time was 30 s. The thermal shift for HRM consisted of five steps: denaturation for 10 s at 95 °C; annealing for 1 min at 60 °C, then raising the temperature at a rate of 0.015 °C/s for denaturation and fluorescence data acquisition; 15 s at 95 °C, and 15 s at 60 °C. Melting profiles of PCR amplicons were obtained with the ViiA7 real-time PCR system software, v. 1.2.2 (Life Technologies). To optimize the DNA pooling conditions, HRM analysis of the Ln_ex1 region of Glyma20g25000 (380 bp, Additional file 2: Table S2) was performed using mixtures of the wild-type gene and mutated gene (Asp9His) at various ratios: 1:1, 1:2, 1:3, 1:4, 1:5, 1:6, 1:7, 1:8, 1:9, 1:10, 1:11, 1:12, 1:13, 1:14, 1:15, and 1:16. The 1:1 to 1:8 mixtures allowed us to clearly distinguish between the mutant profile and the wild-type profile based on the difference plot generated by the software; therefore, the most suitable pooling condition to identify heterozygous mutants was determined to be four samples (eight copies in haploids). For mutation screening, 384 DNA pools (four samples per pool) from 1536 M2’ plants were used. The initial screening for DNA pools containing mutations was repeated at least three times. Then, four DNA samples from the pool in which a mutation was detected were sequenced separately to determine the mutated sequence. The PCR products for sequencing were amplified by using GoTaq® Colorless Master Mix as above and cleaned with ExoSAP-IT (USB Corporation, Cleveland, OH, USA). Sequencing was performed with an ABI Prism BigDye terminator v 3.1 cycle sequencing kit (Applied Biosystems) and 5 pmol of the primer on an ABI-3730xl automated DNA analyzer (Applied Biosystems) according to the manufacturer’s manual. The sequence chromatograms were aligned to the reference sequence with Sequencher 5.2 (Gene Codes Corporation, Ann Arbor, MI, USA) and the difference between wild-type Enrei and the mutant was identified.

### Indexed amplicon sequencing

Long-range PCR (1.3–7.5 kb) was conducted to amplify seven genes: Glyma05g01770, Glyma06g19820, Glyma06g23026, Glyma08g46520, Glyma11g15580, Glyma20g22160, and Glyma20g25000 (see Additional file 2: Table S3 and Additional file 1: Figure S2 for primer pairs). PCR reaction mixtures (10 μl) contained 0.2 μl of template DNA from each of the 384 DNA pools described above, 2 μl of 5× PrimeSTAR GXL Buffer (Takara Bio, Shiga, Japan), 1.0 μl of PrimeSTAR GXL DNA Polymerase (1.25 U/μl), 0.8 μl of 2.5 mM dNTPs, and 0.1 μl of 20 μM forward and reverse primers. PCR reactions were performed on a GeneAmp PCR System 9700 (Applied Biosystems) with the following programs: for Glyma05g01770 and Glyma06g19820, initial denaturation for 5 s at 98 °C; 30 cycles of denaturation for 10 s at 98 °C, annealing for 15 s at 68 °C, and extension for 7 min 35 s at 68 °C; and final extension for 30 s at 68 °C; for Glyma08g46520, Glyma06g23026, and Glyma20g25000 the extension time was decreased to 2 min 30 s, and the annealing temperature for these three genes and for Glyma20g22160 and Glyma11g15580 was decreased to 60 °C. Single banding pattern of PCR amplicons from several rows of a 384 well plate were visually confirmed by 1 % agarose gel electrophoresis and quantified with a Quant-iT™ PicoGreen dsDNA assay kit (Invitrogen). According to the amplicon sizes and approximate concentrations, PCR amplicons of seven genes were combined in equimolar amounts keeping a 384-well format. Four of the combined 384 samples were mixed as described in Fig. 2 to prepare 96 PCR amplicon pools. The pools were purified by ethanol precipitation and were diluted to 0.2 ng/μl. A DNA library for sequencing was prepared by using the transposome-based Nextera™ XT DNA Sample Preparation Kit and Nextera™ XT Index Kit (Illumina). The 96 samples were indexed by using 12 different i7 and eight different i5 adaptors, and mixed. Paired-end sequencing (250 bp) was performed on a MiSeq™ platform using a MiSeq™ v2 500-cycle kit (Illumina) with default parameters. Fluorescent images were analyzed with the MiSeq™ Control software, and FASTQ-formatted sequence data after demultiplexing was created by using MiSeq™ Reporter software. Read mapping and variant detection were undertaken with the CLC Genomics Workbench software using a workflow tool and batch processing of 96 samples. The parameters for sequence trimming and read mapping were the same as for the detection of mutations by whole-genome re-sequencing. Cleaned paired-end reads were mapped onto the target reference sequences. Sequences at polymorphic sites between the donor of the reference sequence and the donor of the mutant library (between Williams 82 and Enrei) were replaced with the sequence of the mutant library donor Enrei in advance to the read mapping. SNVs were identified with quality variant detection modules using the following parameters: ignore non-specific matches and broken read pairs, neighborhood radius five, maximum gap and mismatch count two, minimum neighborhood quality 15, minimum central quality 20, maximum expected alleles four, minimum read coverage 96, minimum variant frequency 2.1 %, and presence in both forward and reverse reads required. The combination of the minimum read coverage 96 and variant frequency 2.1 % parameters corresponded to two reads with a base change among 96 reads per pool. Insertions and deletions were excluded from a analysis. Finally, the mutations predicted to change amino acids were analyzed by using the annotated version of Gmax_189.

### Availability of supporting data

The raw sequence reads for all mutant lines are available under DDBJ (DNA Data Bank of Japan) Sequence Read Archive accession number 'DRA004035-DRA004046' (http://trace.ddbj.nig.ac.jp/DRASearch).

## Additional files

Additional file 1: Figure S1. Regions of Glyma20g25000 scanned by using HRM analysis and indexed amplicon sequencing. Yellow, coding regions; green, transcripts; red, regions scanned by using HRM analysis; purple, the region indexed by using amplicon sequencing analysis. The labels indicate either the gene name (Glyma) or target regions in Additional file 2: Table S2 and Table S3. **Figure S2.** Structures of seven genes analyzed by using indexed amplicon sequencing. Yellow, coding regions; green, transcripts; red, primer binding sites. The labels indicate either gene names (Glyma) or primer names. **Figure S3.** Variation of read coverage by sample well location. (PPTX 272 kb) Additional file 2: Table S1. Soybean simple sequence repeat markers. **Table S2.** Primer pairs for high resolution melting analysis. **Table S3.** Primer pairs for amplicon sequencing. (DOCX 33 kb)

## Abbreviations

EMS: Ethyl methanesulfonate
NGS: Next generation sequencing
HRM: High resolution melting
SSR: Simple sequence repeat
SNV: Single nucleotide variants
NIAS: National Institute of Agrobiological Sciences
TILLING: Targeting-induced local lesions in genomes
