# Supplementary material for: Construction of a high-density mutant library in soybean and development of a mutant retrieval method using amplicon sequencing
Source: BMC Genomics. 2015 Nov 26;16:1014. doi: 10.1186/s12864-015-2079-y (PMC4662035; doi:10.1186/s12864-015-2079-y)
Supplement: Additional file 1: Figure S1. — Regions of Glyma20g25000 scanned by using HRM analysis and indexed amplicon sequencing. Yellow, coding regions; green, transcripts; red, regions scanned by using HRM analysis; purple, the region indexed by using amplicon sequencing analysis. The labels indicate either the gene name (Glyma) or target regions in Additional file 2: Table S2 and Table S3. Figure S2. Structures of seven genes analyzed by using indexed amplicon sequencing. Yellow, coding regions; green, transcripts; red, primer binding sites. The labels indicate either gene names (Glyma) or primer names. Figure S3. Variation of read coverage by sample well location. (PPTX 272 kb) [file 12864_2015_2079_MOESM1_ESM.pptx]

## Slide 1
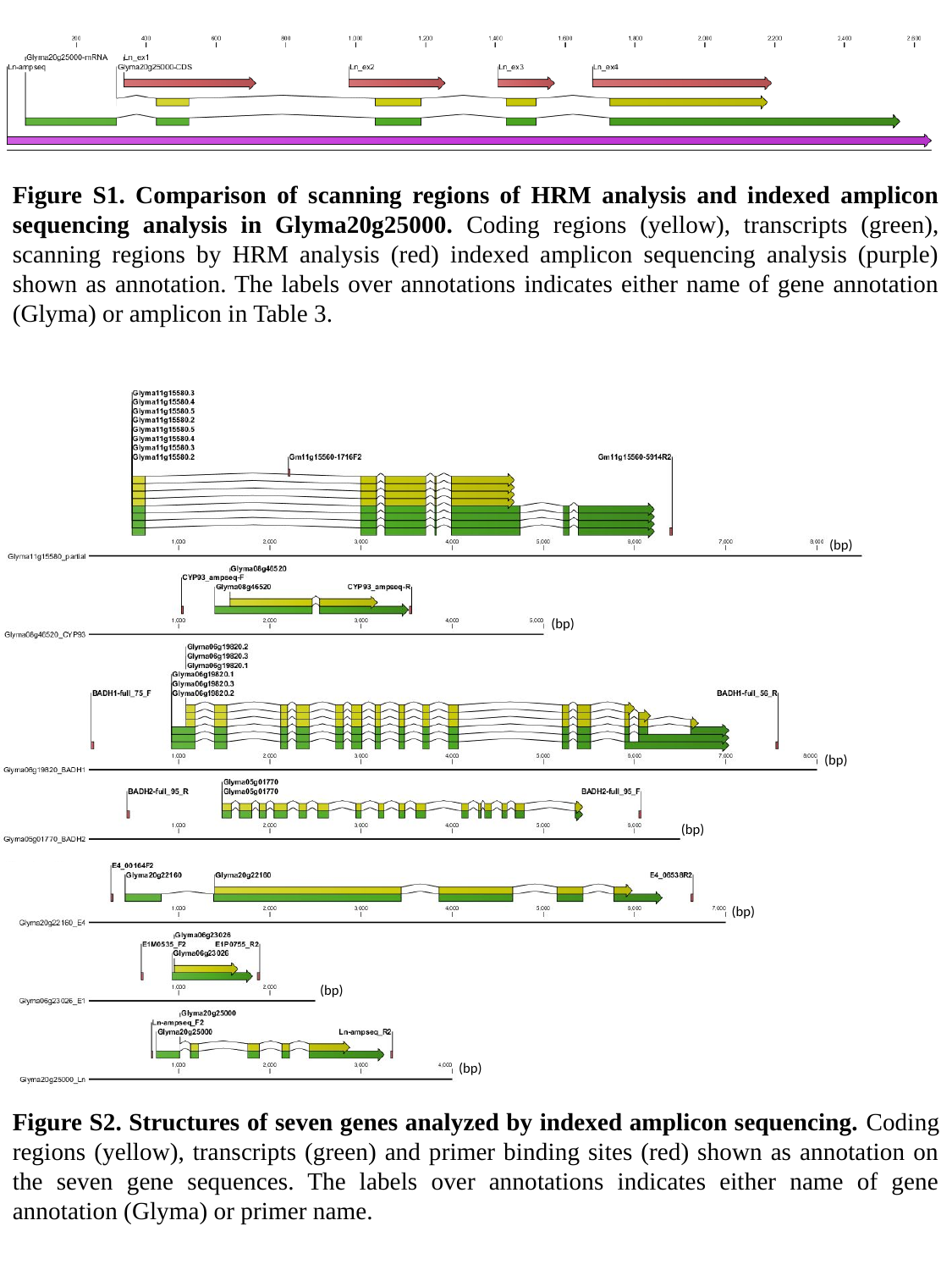

Figure S1. Comparison of scanning regions of HRM analysis and indexed amplicon sequencing analysis in Glyma20g25000. Coding regions (yellow), transcripts (green), scanning regions by HRM analysis (red) indexed amplicon sequencing analysis (purple) shown as annotation. The labels over annotations indicates either name of gene annotation (Glyma) or amplicon in Table 3.
(bp)
(bp)
(bp)
(bp)
(bp)
(bp)
(bp)
Figure S2. Structures of seven genes analyzed by indexed amplicon sequencing. Coding regions (yellow), transcripts (green) and primer binding sites (red) shown as annotation on the seven gene sequences. The labels over annotations indicates either name of gene annotation (Glyma) or primer name.

## Slide 2
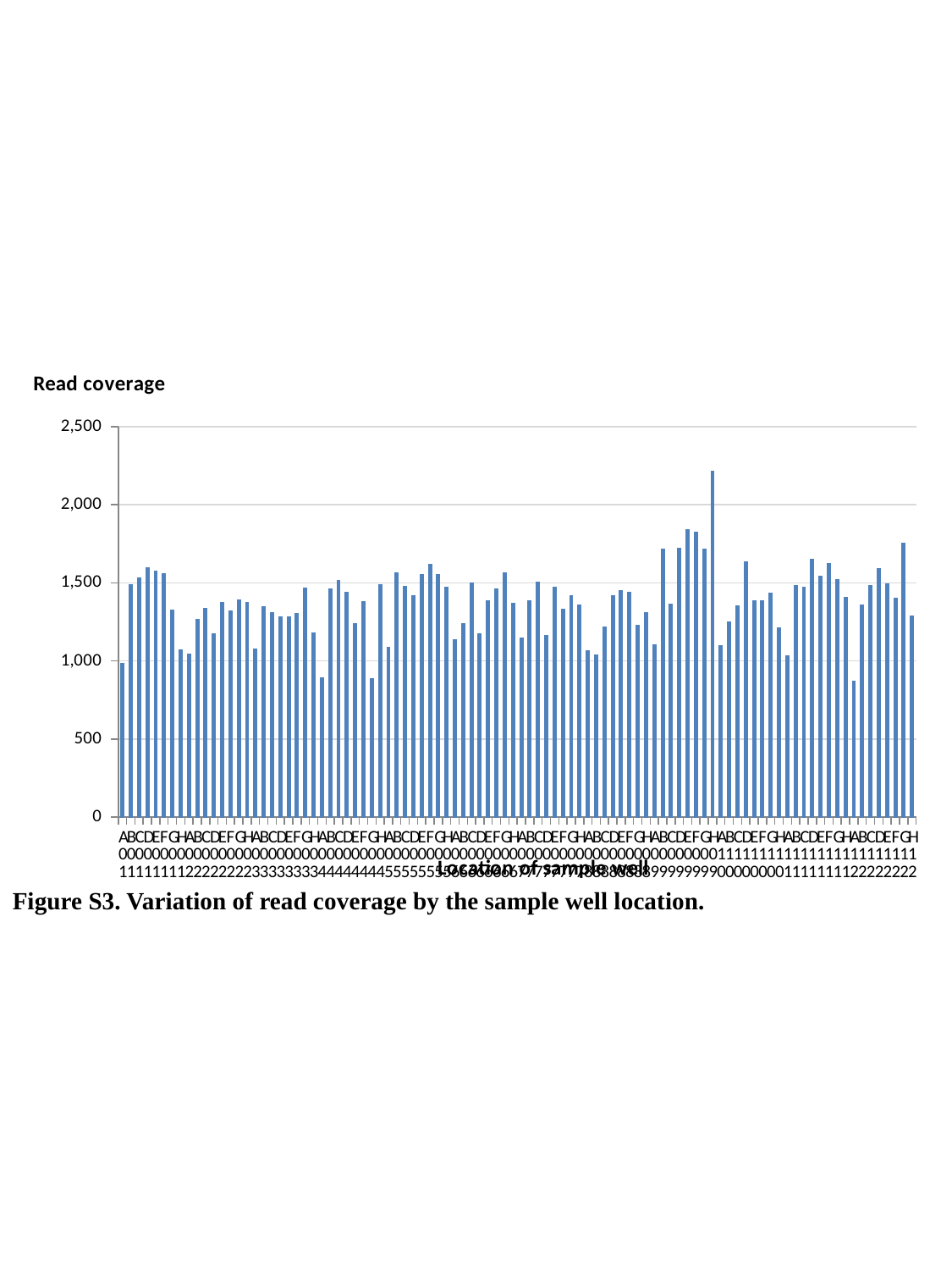

### Chart
| Category | Read coverage |
|---|---|
| A01 | 983.6119792527007 |
| B01 | 1485.951435462024 |
| C01 | 1531.3929763123988 |
| D01 | 1593.9243120023787 |
| E01 | 1576.4270706002842 |
| F01 | 1559.7721100796193 |
| G01 | 1326.6012752320855 |
| H01 | 1072.2386269780964 |
| A02 | 1041.854075126367 |
| B02 | 1268.2325811886749 |
| C02 | 1336.7431035052364 |
| D02 | 1173.2170867884633 |
| E02 | 1373.9825233737488 |
| F02 | 1320.9919059103372 |
| G02 | 1390.6897816247647 |
| H02 | 1375.3745416102283 |
| A03 | 1075.7325977072253 |
| B03 | 1344.395321946546 |
| C03 | 1310.9913112425254 |
| D03 | 1280.9905513892102 |
| E03 | 1281.4862070104728 |
| F03 | 1302.467904456705 |
| G03 | 1467.160989791536 |
| H03 | 1180.0437080841784 |
| A04 | 889.9862896032245 |
| B04 | 1462.55522151376 |
| C04 | 1512.976807955334 |
| D04 | 1439.7650071029766 |
| E04 | 1238.3721959760812 |
| F04 | 1382.1375334500644 |
| G04 | 884.9183653242592 |
| H04 | 1485.851795566421 |
| A05 | 1085.5625557501073 |
| B05 | 1562.3932406092042 |
| C05 | 1475.8587003204598 |
| D05 | 1417.5977732994152 |
| E05 | 1553.0607552281213 |
| F05 | 1620.3728567180945 |
| G05 | 1550.5207307806668 |
| H05 | 1470.18457828141 |
| A06 | 1135.0737718457829 |
| B06 | 1238.946810267931 |
| C06 | 1501.1615514222472 |
| D06 | 1175.5213584855794 |
| E06 | 1383.1015230103405 |
| F06 | 1462.3006376160429 |
| G06 | 1562.542039710595 |
| H06 | 1370.7025009085203 |
| A07 | 1144.0119594304404 |
| B07 | 1384.3449073309325 |
| C07 | 1504.0397766691995 |
| D07 | 1160.0829231226667 |
| E07 | 1470.0330040635633 |
| F07 | 1330.7969870164195 |
| G07 | 1417.7532789322408 |
| H07 | 1358.4862070104728 |
| A08 | 1064.8564868347155 |
| B08 | 1036.12636691004 |
| C08 | 1216.6880306584294 |
| D08 | 1419.7777924609336 |
| E08 | 1449.0080280154614 |
| F08 | 1439.9539462816742 |
| G08 | 1226.2430209124848 |
| H08 | 1307.6613036439921 |
| A09 | 1101.188807030295 |
| B09 | 1715.502196967194 |
| C09 | 1362.940500181704 |
| D09 | 1721.6487825828406 |
| E09 | 1842.0784300769765 |
| F09 | 1821.2322838547689 |
| G09 | 1717.810994747101 |
| H09 | 2212.2781393504906 |
| A10 | 1096.970002312597 |
| B10 | 1248.644025240345 |
| C10 | 1352.587168390102 |
| D10 | 1634.0458885328223 |
| E10 | 1386.4303082361491 |
| F10 | 1385.906868413228 |
| G10 | 1433.4138557600186 |
| H10 | 1213.7863490700056 |
| A11 | 1034.4789718854274 |
| B11 | 1483.0808748224256 |
| C11 | 1473.3940004625194 |
| D11 | 1649.6174964485117 |
| E11 | 1543.7394363870626 |
| F11 | 1622.3378373913906 |
| G11 | 1522.4542601341307 |
| H11 | 1405.3856090389506 |
| A12 | 870.8429085863424 |
| B12 | 1360.4568370279824 |
| C12 | 1484.091843139846 |
| D12 | 1589.5747464402525 |
| E12 | 1494.9741649872808 |
| F12 | 1401.0042617859856 |
| G12 | 1750.8928276454458 |
| H12 | 1288.792427896528 |Figure S3. Variation of read coverage by the sample well location.
