# Supplementary material for: Construction of a high-density mutant library in soybean and development of a mutant retrieval method using amplicon sequencing
Source: BMC Genomics. 2015 Nov 26;16:1014. doi: 10.1186/s12864-015-2079-y (PMC4662035; doi:10.1186/s12864-015-2079-y)
Supplement: Supplementary file 2 [file 12864_2015_2079_MOESM2_ESM.docx]

Table S1. Soybean simple sequence repeat markers.

Multiplex	Fluorescent dye	Fragment size in seed parent (bp)	Fragment size of unexpected product (bp)
A	NED	347	350
B	NED	348	No variation
A	FAM	274	No variation
B	HEX	258	245, 250, 254, 262
B	FAM	200	191, 197, 205, 217
A	HEX	232	No variation
B	NED	307	294, 312, 327
A	HEX	334	338
B	HEX	356	352, 360, 378
B	FAM	278	280
A	NED	210	No variation
A	NED	201	204
B	HEX	148	152, 166
B	FAM	180	160, 165

Table S2. Primer pairs for high resolution melting analysis.

Gene locus	Amplicon size (bp)	Primer name	Primer sequence	Length	Chromosome	Start position of primer (bp)	End position of primer (bp)	Region name	Region size (bp)	Start position (bp)	End position (bp)
Glyma20g25000	380	Ln_ex1_F	CTACACCTTCACACCCTTCTTTTCT	25	Gm20	34,688,534	34,688,558	Ln_ex1	332	34,688,534	34,688,913
		Ln_ex1_R	CAAGGAATATACCACAGGGATGG	23	Gm20	34,688,913	34,688,891				
	277	Ln_ex2_F	GGTAATCGAGAATGGGGAGTT	21	Gm20	34,689,179	34,689,199	Ln_ex2	231	34,689,179	34,689,455
		Ln_ex2_R	CTTGTGAGAAGGAGTGGTAAAAGAA	25	Gm20	34,689,455	34,689,431				
	163	Ln_ex3_F	GTTTTTGTGATTGTTTTGAGCAG	23	Gm20	34,689,606	34,689,628	Ln_ex3	114	34,689,606	34,689,768
		Ln_ex3_R	CAAGAAGAAGAAATAGGAAGAGAATC	26	Gm20	34,689,768	34,689,743				
	514	Ln_ex4_F	CATGACACTGTTTGATACTGCGA	23	Gm20	34,689,877	34,689,899	Ln_ex4	467	34,689,877	34,690,390
		Ln_ex4_R	CTCTCATCTCTTTAGAAACCATCT	24	Gm20	34,690,390	34,690,367				
Glyma08g46520	479	CYP93_HRM-F3	AATGGGGAAGAAGAGTAATGCGGAAAAC	28	Gm08	45,602,873	45,602,846	CYP93E1-1	429	45,602,395	45,602,873
		CYP93_HRM-R3	CAGCTCCGCCAGTGACCACTCT	22	Gm08	45,602,395	45,602,416				
	98	CYP93_Gln437-F1	AGCTTCTTCTCCCGCTCC	18	Gm08	45,602,013	45,602,030	CYP93E1-2	58	45,602,013	45,602,110
		CYP93_Gln437-R1	GGAGAGGTTCTTGTTCTCCGAC	22	Gm08	45,602,110	45,602,089				
Glyma06g23026	344	Gm06E1as_till1_ned_F	CACTCAAATTAAGCCCTTTC	20	Gm06	20,007,039	20,007,058	Gm06E1	305	20,007,039	20,007,382
		Gm06E1as_till1_fam_R	GTTTCTTCACCAAATCTGC	19	Gm06	20,007,382	20,007,364				
Glyma11g15580	120	Gm11_11232087_2104_G_del_F	GATAGTAAGTGTGCTCGACTTGG	23	Gm11	11,234,132	11,234,154	Gm11_11232087_2104_G_del	71	11,234,132	11,234,251
		Gm11_11232087_2104_G_del_R	AGAAGAATAACATCTGTTCATCTCAG	26	Gm11	11,234,251	11,234,226				
	131	Gm11_11232087_2187_G_A_F	TCCCGGGCAGTAGTTT	16	Gm11	11,234,206	11,234,221	Gm11_11232087_2187_G_A	95	11,234,206	11,234,336
		Gm11_11232087_2187_G_A_R	GGCACCAAACTTGATACTTT	20	Gm11	11,234,336	11,234,317				
	202	Gm11_11232087_2892_A_T_F	CAGCCTTTCCAAAGTTAGTTCAA	23	Gm11	11,234,860	11,234,882	Gm11_11232087_2892_A_T	156	11,234,860	11,235,061
		Gm11_11232087_2892_A_T_R	AGGACAACTGGAATACAATGGTG	23	Gm11	11,235,061	11,235,039				
	99	Gm11_11232087_3028_G_A_F	CAGGCATTGCTCTGTCTTT	19	Gm11	11,235,068	11,235,086	Gm11_11232087_3028_G_A	58	11,235,068	11,235,166
		Gm11_11232087_3028_G_A_R	ACTCAAGGAACTGTCCTGCAAT	22	Gm11	11,235,166	11,235,145				
Glyma20g22160	260	E4_1770_190-1_36_F	GGCAGTTGTAGTCAATGACAATGAAG	26	Gm20	32,089,409	32,089,434	E4-CDS1-1	211	32,089,409	32,089,668
		E4_1770_190-1_36_R	CATCTCGCATCACCAGATCACAC	23	Gm20	32,089,668	32,089,646				
	267	E4_2260_179-1_71_F	TTGTGGAATGGCAGCAGTTAGAGTA	25	Gm20	32,089,901	32,089,925	E4-CDS1-3	217	32,089,901	32,090,167
		E4_2260_179-1_71_R	TCCATACTCTCGGTATCTTTGAATG	25	Gm20	32,090,167	32,090,143				
	284	E4_3374_175-1_29_F	ACAAATTTACGCGTATTGAAGGTGA	25	Gm20	32,091,019	32,091,043	E4-CDS2-1	234	32,091,019	32,091,302
		E4_3374_175-1_29_R	AACCTTCTCTGTTTCCAAACCAGTC	25	Gm20	32,091,302	32,091,278				
	378	E4_3722_280-2_62_F	CCTAGTTACTGGGGTCTTCTGCTTC	25	Gm20	32,091,380	32,091,404	E4-CDS2-3	327	32,091,380	32,091,757
		E4_3722_280-2_62_R	CTTGAGTTTCAATGAGGTTTCAACTG	26	Gm20	32,091,757	32,091,732				
	248	E4_4524_190-1_33_F	CTGCATGAAGTTCTGGTTACCTCAC	25	Gm20	32,092,180	32,092,204	E4-CDS3-1	198	32,092,180	32,092,427
		E4_4524_190-1_33_R	TTAACGAGATGGACGGATTTGCCTA	25	Gm20	32,092,427	32,092,403				

Table S3. Primer pairs for amplicon sequencing.

Gene locus	Amplicon size (bp)	Primer name	Primer sequence	Length	Chromosome	Start position of primer (bp)	End position of primer (bp)	Region name	Region size (bp)	Start position (bp)	End position (bp)
Glyma05g01770	5,643	BADH2-full_95_F	TGCTATCCCATTAAACACCCACACCTTGAGAT	32	Gm05	1,232,425	1,232,456	BADH2-full	5,580	1,232,425	1,238,067
		BADH2-full_95_R	GCAAGTGTAATCAAGTTGGTTTAGATTCGAT	31	Gm05	1,238,067	1,238,037				
Glyma06g19820	7,539	BADH1-full_75_F	TATTATACACTCAGCCAGGATTTCTCGGCAAC	32	Gm06	16,161,041	16,161,072	BADH1-full	7,475	16,161,041	16,168,579
		BADH1-full_56_R	ACTAGACTTCATATCCCTCATTGGTGAGACCT	32	Gm06	16,168,579	16,168,548				
Glyma06g23026	1,304	E1M0535_F2	TTAATCCCGTTTGATTGGTTTTTGGT	26	Gm06	20,006,589	20,006,614	E1	1,248	20,006,589	20,007,892
		E1P0755_R2	AGGTTTCAACCGTGCATAGATCTCCTGCTA	30	Gm06	20,007,892	20,007,863				
Glyma08g46520	2,531	CYP93_ampseq-F	ACATGTTAGGTGATCCGACATTCTGTGAAA	30	Gm08	45,603,974	45,603,945	CYP93_ampseq	2,469	45,601,444	45,603,974
		CYP93_ampseq-R	GGACTTTTAAACAGAGAGCACAATTATGAATC	32	Gm08	45,601,444	45,601,475				
Glyma11g15580	4,214	Gm11g15560-1716F2	TGGTGTCCTTGGTTGGAGTGGAGCAC	26	Gm11	11,236,291	11,236,266	Gm11g15560	4,155	11,232,078	11,236,291
		Gm11g15560-5914R2	ACAATTCTTGCTAATAAAATTAACTATCATCAC	33	Gm11	11,232,078	11,232,110				
Glyma20g22160	6,390	E4_00164F2	CTTTTTCACGTAGATTCTCCTAACAC	26	Gm20	32,087,258	32,087,283	E4	6,334	32,087,258	32,093,647
		E4_06538R2	ACTCTAATCCTCTGTGCCAAACATATTCCG	30	Gm20	32,093,647	32,093,618				
Glyma20g25000	2,648	Ln-ampseq_F2	CCTCCACACTCACTCTCACA	20	Gm20	34,688,200	34,688,219	Ln-ampseq	2,603	34,688,200	34,690,847
		Ln-ampseq_R2	GGGGTTTTAGAGATTTTGGAATAGC	25	Gm20	34,690,847	34,690,823				
